# Performance analysis of a machine learning flagging system used to identify a group of individuals at a high risk for colorectal cancer

**DOI:** 10.1371/journal.pone.0171759

**Published:** 2017-02-09

**Authors:** Yaron Kinar, Pinchas Akiva, Eran Choman, Revital Kariv, Varda Shalev, Bernard Levin, Steven A. Narod, Ran Goshen

**Affiliations:** 1 Medial Research, Kfar Malal, Israel; 2 Medial EarlySign, Kfar Malal, Israel; 3 Department of Gastroenterology, Souraski Medical Centre, Tel-Aviv, Israel; 4 Faculty of Medicine, Tel Aviv University, Tel-Aviv, Israel; 5 Institute of Health Research and Innovation, Maccabi Healthcare Services, Tel-Aviv, Israel; 6 Dalla Lana School of Public Health, University of Toronto, Toronto, Canada; 7 Women’s College Research Institute, Toronto, Canada; University of Munich, GERMANY

## Abstract

Individuals with colorectal cancer (CRC) have a tendency to intestinal bleeding which may result in mild to severe iron deficiency anemia, but for many colon cancer patients hematological abnormalities are subtle. The fecal occult blood test (FOBT) is used as a pre-screening test whereby those with a positive FOBT are referred to colonscopy. We sought to determine if information contained in the complete blood count (CBC) report coud be processed automatically and used to predict the presence of occult colorectal cancer (CRC) in the setting of a large health services plan. Using the health records of the Maccabi Health Services (MHS) we reviewed CBC reports for 112,584 study subjects of whom 133 were diagnosed with CRC in 2008 and analysed these with the MeScore tool. The odds ratio for being diagnosed with CRC in 2008 was calculated with regards to the MeScore, using cutoff levels of 97% and 99% percentiles. For individuals in the highest one percentile, the odds ratio for CRC was 21.8 (95% CI 13.8 to 34.2). For the majority of the individuals with cancer, CRC was not suspected at the time of the blood draw. Frequent use of anticoagulants, the presence of other gastrointestinal pathologies and non-GI malignancies were assocaitged with false positive MeScores. The MeScore can help identify individuals in the population who would benefit most from CRC screening, including those with no clinical signs or symptoms of CRC.

## Introduction

Early detection of colorectal cancer through active screening has been shown in several studies to reduce mortality [1,2]. Classical screening methods include direct visualization and sampling through colonoscopy or sigmoidoscopy or indirectly through the detection of occult blood in the feces. The latter screening method is based on the tendency for many CRCs to bleed slowly and persistently and this may lead to a state of iron deficiency anemia. Among elderly men and women, one of the most common causes of iron deficiency anemia is CRC [3]. Iron deficiency anemia is diagnosed through the interpretation of CBC, which is a compendium of 20 hematologic parameters, including hemoglobin level and the mean volume and distribution of volumes of the red blood cells. It is also possible that some CRCs impact on the various cell fractions within the immune system and that these changes create a characteristic signature of the white blood cells. Similarly, platelets have been implicated in CRC carcinogenesis and progression in several studies, although the exact mechanism by which platelets contribute to CRC incidence and metastasis is not known [4 5]. It is of interest to determine whether or not CBC reports, used singly or in series, can be scanned automatically and interpreted using a machine learning based algorithm in order to identify a subgroup of individuals who have a higher than average probability of harboring an occult CRC. The information content of the CBC report could potentially be enhanced by incorporating measures of change across time as well as absolute levels. If so, we could use CBC reports as a passive means of CRC case identification and thereby identify candidates for additional investigations, such as colonoscopy. We have previously developed a machine learning-based algorithm (MeScore) to predict the occurrence of CRC based on the information contained in the CBC report [6]. In the present study, we evaluate the performance of the MeScore algorithm, using a large cohort of individuals at average risk of CRC. We generated a risk score for each member of a cohort of individuals at risk for colon cancer, evaluated whether or not they developed colon cancer by linkage to the Israel Cancer Registry and we reviewed the charts of all patients diagnosed with CRC and a sample of false positives.

## Methods

**Fig 1:**
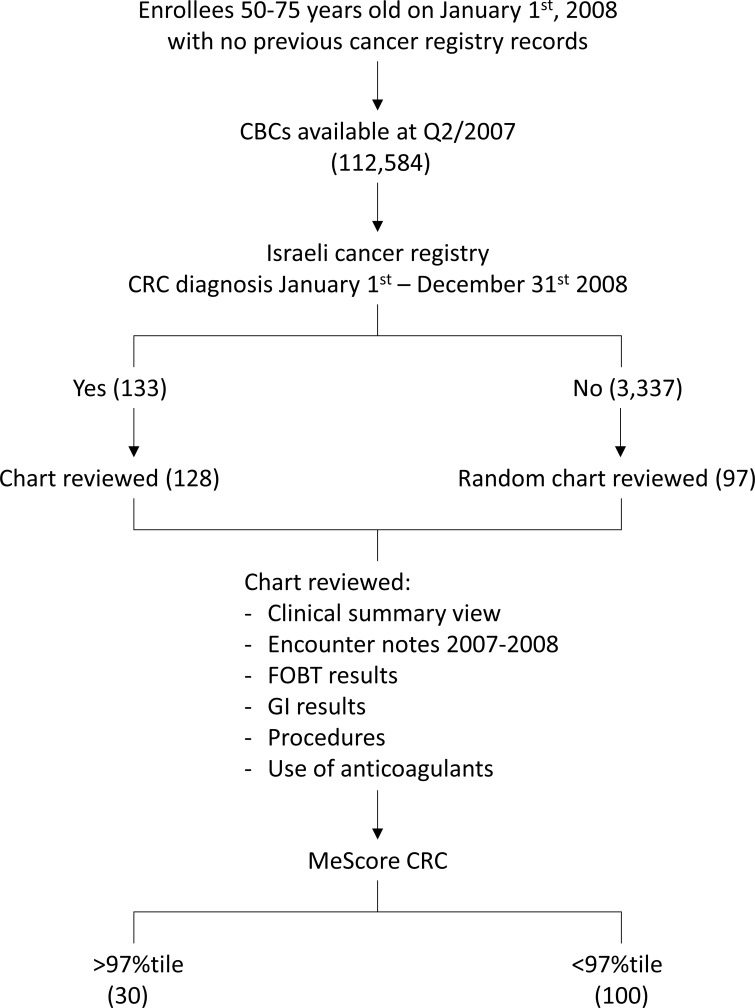
Schema of the study design.

This analysis is based on reviewing the demographics and CBC tests results derived from MHS electronic medical records and linking these data to the records of the Israel Cancer Registry (Fig 1). MHS is the second largest health care provider in Israel with approximately two million enrollees. The study was approved by MHS ethical committee (32/2016) and designed as follows: we defined the testing period as the six-month interval from July 1, 2007 to December 31, 2007. We considered the test cohort to be all men and women between ages 50 and 75 on January 1, 2008 who had one or more CBC report recorded in the MHS electronic medical record system for a blood sample taken during the six month testing period. This CBC report was called the index report. Subjects included in the study were de-identified and therefore participant consent was not required. This consent procedure was approved by the MHS Ethical Committee.

Subjects were excluded if they had a diagnosis of CRC or any other cancer recorded in the National Cancer Registry prior to January 1, 2008 or if they had no index blood test taken during the testing period. In general, a CBC is done as part of the medical work-up to all individuals over the age of 50 and approximately 75% of MHS enrollees have a CBC within a given year. In the event that there were two or more CBC reports listed for the testing period, the last dated report was the index report. Each study subject was assigned a score from 1 to 100, based on the information contained in the CBC report using a validated computational scoring algorithm (MeScore, with a minimal score of 18 at age 50), which has been described in detail previously [6]. The algorithm incorporates information on the subjects age and sex as well as on all available CBC reports, including the index CBC report and all prior reports from January 2003 onwards. The model uses all the analyte values contained in the CBC report (including the most recent report and past reports), as well as age and sex, in an ensemble of decision trees [7] and gradient boosting machines [8]. Using the algorithm, a score was generated for each individual on the date of the index report. The scores were then transformed into percentile values. Results are presented in terms of percentiles (and not the raw scores).

Using a record linkage approach, we sought to establish which study subjects were diagnosed with CRC in the one-year follow-up period from January 1, 2008 to December 31, 2008 (the diagnostic period). Record linkage was performed by MHS staff using the unique national identification number. The date of diagnosis was defined by the Israel National Cancer Registry to be the date of biopsy.

There were 133 patients for whom CRC was diagnosed in the one-year diagnostic period (2008) according to the records of the Israel National Cancer Registry. The electronic medical records of 128 patients (96%) were retrieved and reviewed by authorized MHS staff. The review was done to confirm the diagnosis of cancer, to establish if the patient was asymptomatic at diagnosis (screen-detected) or presented with symptoms. Symptoms included evidence for gastrointestinal bleeding, or a change in bowel habits. We also established whether or not anemia was present at the time of diagnosis. Each patient was determined to have anemia based on the clinical standard in place at MHS, based on the hemoglobin value taken prior to diagnosis (>13.5 g/dL for men and >12.5g/dL for women). We also determined if the patient was under investigation for CRC prior to the date of the index blood test, e.g., for some patients, anemia had been noted in the medical records and these patients had undergone a colonoscopy to rule out or to confirm CRC. For other patients, no indication was noted in the medical record that CRC had been suspected at the time of the positive index CBC. For 57 of the 133 CRC patients the result of a FOBT was recorded in the medical record in the two-year period prior to index report (43%). For 14 of these patients the result of the FOBT was positive (3/3), 19 partially positive (1 or 2/3), and for 24 of these patients the result of the FOBT was negative.

The primary analysis was based on a cut point of 97% (top 3%ile.) There were 3337 subjects who had a test level above the 97^th^ percentile. In order to determine the clinical parameters associated with a false positive test, we reviewed the medical records of 97 individuals selected at random from among those individuals who had a score above the 97% percentile and who did not have CRC. This was done to ensure that they did not have a cancer that was not recorded by the Israel Cancer Registry and to determine factors that might be associated with a false positive result at the 97%-ile level. We flagged conditions associated with bleeding, such as anemia, the use of aspirin or other anticoagulants, symptoms of gastrointestinal conditions and other cancers diagnosed during the index year. We classified each of the 97 controls as to whether or not they were referred for a GI consultation within MHS or if significant gastrointestinal findings were identified through the MHS system. We also reviewed the results of a sample of 97 controls who had a MeScore level in the normal range for the prevalence of aspirin use, gastrointestinal illness and cancer.

For the purpose of evaluating the performance of the percentile score as a pre-screening test, we considered two cut points for a positive result, which corresponded to scores in the top three percentile (score = 97.02) and in the top one percentile (score = 99.38). In this way, each patient was classified twice, according to the score (normal or abnormal with regard to the two chosen cut points) and CRC status (cancer, no cancer). We estimated sensitivity, yield (positive predictive value) and the odds ratios for being diagnosed with CRC within the diagnostic period, given an abnormal score combined with calculation of 95% confidence interval based on bootstrapping analysis. Both crude and adjusted odds ratios were generated. We repeated the analyses for patient subgroups defined by sex, and for time from index CBC to cancer diagnosis. We also identified additional cases of cancer in the extended follow up period until January 1, 2013.

## Results

There were 112,584 study subjects eligible for the study who were alive and without CRC on January 1, 2008. Each of these subjects had an index CBC report recorded within the six month testing period. The average age of the study subjects on January 1^,^ 2008 was 60.9 years (range 50 to 75 years); 62,785 (56%) were female and 49,799 (44%) were male. There were 133 cases of CRC diagnosed during 2008 (118 per 100,000 per annum). The incidence was 100 per 100,000 among women in the cohort and 140 per 100,000 among men. The mean MeScore was 59.3 (range 22.24 to 100.00) for males and was 46.8 (range 17.07 to 99.99) for females. Of the 112,584 study subjects, 3337 and 1094 were assigned a score as positive using the top three percentile cutoff (score > 97.02) and the top one percent cutoff (score > 99.38) points, respectively. The two analyses are presented separately below in Table 1.

**Table 1:** Test yield and sensitivity at 1% percentle and 3% percentile cutoff values for cancers diagnosed in the diagnostic period.

Positive Rate	1%	3%
Number of subjects with abnormal scores	1094	3337
Cancer cases within the diagnostic period using cutoff	23	33
Total cancer cases	135	135
Sensitivity	17.3%	24.8%
Yield	2.1%	1.0%
Odds Ratio (95% CI)	21.8 (13.8–34.2)	10.9 (7.3–16.2)

### Three percentile cutoff

Among the 3337 individuals with a positive test, there were 33 CRCs diagnosed in 2008 (yield 1.0%). Among the 109,247 individuals with a negative test, there were 100 CRCs diagnosed (0.09%). The odds ratio for CRC given a positive test was 10.9 (95% CI 7.3 to 16.2). Overall, the test sensitivity was 25%. The sensitivity was 29% for cancers diagnosed in the first six months of 2008 and was 20% for the latter six months of 2008.

### One percentile cutoff

Among the 1094 individuals with a positive test, there were 23 CRCs diagnosed in 2008 (yield 2.1%). Among the 111,490 individuals with a negative test, there were 110 CRCs diagnosed (0.09%). The odds ratio for CRC, given a positive test was 21.8 (95% CI 13.8 to 34.2). Overall, the test sensitivity was 17.3%. The sensitivity was 25% for cancers diagnosed in the first six months of 2008 and was 9.5% for the second six months of 2008.

Of the 133 individuals with CRC, there were 70 males and 63 females. The average age of diagnosis was 64.5 years. Charts were reviewed for 128 of the 133 CRC patients. For 84 of the 128 patients (66%), there was no indication in the chart that CRC was suspected on the date of the index blood. 44 of the patients (34%) were under investigation for symptoms of CRC on the date of the index blood test.

### Survey of controls

Using the three percentile cutoff, we identified 3326 individuals with a positive test, of whom 3198 did not have CRC (false positives). We selected a random sample of 97 individuals for chart review (3%). Of these, 67 were taking an anticoagulant (69%); 7 were treated for a gastrointestinal illness (7%) and four (4%) had another cancer diagnosed in 2008. There were 19 controls (20%) with no apparent reason for an abnormal CBC test. Retrieval of data of the charts of the 112,584 eligible documented the use of the anticoagulants in 22% (not age/gender matched to positive tests population).

## Discussion

In this study, we show that MeScore's automated interpretation of the CBC report can potentially be used as a tool to identify individuals who are at 10 to 20 times increased risk of harboring an occult CRC and who are candidates for screening colonoscopy. In a previous paper, the model was developed using a mixed cohort/case-control design, incorporating both patients from Israel and the UK [6]. In the present study, we evaluate this model using the prospective data previously collected and we are able to evaluate the sensitivity and the yield. The CBC-based algorithm was able to discriminate between individuals who did and who did not develop CRC at a high level of statistical significance, using conventional measures of association; individuals in the highest one percentile of the score faced a 20fold risk of CRC in the period from 12 to 18 months post-test (Table 1) but the risk ratio declined in subsequent years (Table 2). Using the one percent cutoff level, the sensitivity declined from 25% in the first half of the diagnostic year to 9.5% in the second half of the diagnostic year. We also evaluated the test using a cutoff of 3%. Using that cutoff level resulted in a sensitivity of 25%; that is 25% of the CRCs in the population were identified within the three percent of the population with the highest score (corresponding to an odds ratio of 10.9). These data suggest that using a one percentile cutoff at six monthly intervals and a three percentile cutoff annually may yield equivalent benefits, provided no stage shift is documented between the two in future studies.

**Table 2:** Cancers diagnosed in cohort from 2008 to 2012 according to test score as determined in 2007.

Risk score	Variable	2008 (n = 112,584)	2009 (n = 111,384)	2010 (n = 110,198)	2011 (n = 108,957)	2012 (n = 107,665)
Colorectal cancers						
1% ile	• Number • Incidence	• 23 • 2040	• 6 • 540	• 3 • 270	• 4 • 367	• 2 • 185
3%ile	• Number • Incidence	• 33 • 975	• 14 • 415	• 9 • 270	• 9 • 249	• 7 • 214
3+%ile	• Number • Incidence	• 100 • 92	• 116 • 107	• 125 • 117	• 152 • 143	• 124 • 118
All	• Number • Incidence	• 133 • 118	• 130 • 117	• 134 • 121	• 161 • 147	• 131 • 122
All cancers						
1% ile	• Number • Incidence	• 50 • 4441	• 22 • 1975	• 15 • 1361	• 9 • 826	• 11 • 1021
3%ile	• Number • Incidence	• 103 • 3019	• 55 • 1629	• 47 • 1407	• 41 • 1242	• 34 • 1042
3+%ile	• Number • Incidence	• 1162 • 1064	• 1073 • 993	• 1080 • 1000	• 1109 • 987	• 1074 • 967
All	• Number • Incidence	• 1265 • 1120	• 1128 • 1013	• 1127 • 1023	• 1150 • 1055	• 1108 • 1029

Incidence: per 100,000 per year.

This implies that if these individuals had been identified through the CBC screen in the year prior to the clinical date of diagnosis, many of them would have been diagnosed in an earlier stage.

Colonoscopy is a sensitive and accurate screening modality, however, given the cost and possible adverse effects, universal screening with colonoscopy is not recommended world-wide as a public health measure. Colonoscopy is generally offered as screening test for people who have a higher than expected prevalence of CRC, including those with a mutation in AFP or one of the mismatch repair genes, those with a positive FOBT, those with family history and predisposing diseases, or a past history of adenomatous polyps. In our study, the CRC incidence of those individuals in the highest three percentile based on the CBC score was about ten times greater than expected. This risk is comparable or higher to the risk for those individuals who have an inherited susceptibility to CRC, but the number of at risk individuals defined by the MeScore is many times greater. In comparison, in a fecal occult blood trial, among those with a positive Hemoccult test, 4.2% were found to have cancer upon confirmatory colonoscopy [9].

The proposed approach is qualitatively different from current practices in that it is passive and opportunistic and carriers little cost. Traditional approaches require active participation of the patients either in the acquisition and processing of a fecal sample or necessitates time-consuming and costly imaging. This is not a replacement for traditional population-based screening programs but provides an additional layer of coverage for individuals enrolled in a health care system such as MHS. We do not suggest here that the CBC analysis can replace FOBT, rather it can be used as an adjunct and might be particularly helpful for the large number of individuals in the population who forego the FOBT in spite of their doctors' recommendation. It is also possible that the abnormal test can lead to the identification of adenomatous polyps which are a precursor of CRC and identifying these has additional value. In the present study we did not have details about the presence of pre-malignant conditions such as colonic polyps. In our study the assessment of CRC status in the cohort was dependent on clinical assessment and the clinician was not aware of the test result. The excess of CRCs in years two and three suggest that many more cancers might have been diagnosed in year one if the test result had been given the physician who then ordered a colonoscopy. This was an observational study and the care of the patient was not altered by a positive test result. Future studies will determine the utility of the CBC test in concert with colonoscopy and would involve assessing a cohort of patients with the CBC test, followed by colonoscopy for those deemed to be at high risk. It is also desirable to improve the risk prediction algorithm using additional markers such as electrolytes and other markers of inflammation and to determine the marginal benefit of a multiple marker based approach over a conventional measure of hemoglobin.

## References

[1] Edwards BK, Ward E, Kohler BA, Eheman C, Zauber AG, Anderson RN, et al Annual report to the nation on the status of cancer, 1975–2006, featuring colorectal cancer trends and impact of interventions (risk factors, screening, and treatment) to reduce future rates. Cancer. 2010; 116(3): 544–73. 10.1002/cncr.24760 19998273 PMC3619726

[2] Giorgi Rossi P, Vicentini M, Sacchettini C, Di Felice E, Caroli S, Ferrari F, et al Impact of Screening Program on Incidence of Colorectal Cancer: A Cohort Study in Italy. Am J Gastroenterol. 2015; 110(9): 1359–66. 10.1038/ajg.2015.240 26303133

[3] Rockey DC and Cello JP. Evaluation of the gastrointestinal tract in patients with iron-deficiency anemia. N Engl J Med 1993; 329: 1691–1695. 10.1056/NEJM199312023292303 8179652

[4] Guillem-Llobat P, Dovizio M, Alberti S, Bruno A, Patrignani P. Platelets, cyclooxygenases, and colon cancer. Semin Oncol 2014; 41(3): 385–96. 10.1053/j.seminoncol.2014.04.008 25023354

[5] Seretis C, Youssef H, Chapman M. Hypercoagulation in colorectal cancer: what can platelet indices tell us? Platelets 2015; 26(2): 114–8. 10.3109/09537104.2014.894969 25192361

[6] Kinar Y, Kalkstein N, Akiva P, Levin B, Half EE, Goldshtein I, Chodick G, Shalev V. Development and validation of a predictive model for detection of colorectal cancer in primary care by analysis of complete blood counts: a binational retrospective study. J Am Med Inform Assoc. 2016; 23(5): 879–90. 10.1093/jamia/ocv195 26911814 PMC4997037

[7] Breiman L. Random forests. Mach Learn 2001; 45(1): 5–32.

[8] Friedman JH. Greedy function approximation: a gradient boosting machine. Ann Statist 2001; 29(5): 1189–232.

[9] Kershenbaum A, Flugelman A, Lejbkowicz F, Arad H, Rennert G. Excellent performance of Hemoccult Sensa in organised colorectal cancer screening. European journal of cancer 2013; 49(4): 923–30. 10.1016/j.ejca.2012.09.020 23099005

